# The Mental Health Outcomes of Drought: A Systematic Review and Causal Process Diagram

**DOI:** 10.3390/ijerph121013251

**Published:** 2015-10-22

**Authors:** Holly Vins, Jesse Bell, Shubhayu Saha, Jeremy J. Hess

**Affiliations:** 1 Department of Environmental Health, Rollins School of Public Health at Emory University, Atlanta, GA 30322, USA; E-Mails: hollyvins@gmail.com (H.V.); jesse.bell@noaa.gov (J.B.); hsf5@cdc.gov (S.S.); 2 Cooperative Institute for Climate and Satellites-NC, Asheville, NC 27695, USA; 3 Department of Emergency Medicine, Emory University School of Medicine, Atlanta, GA 30307, USA

**Keywords:** climate change, human health, drought, mental health, causal process diagram

## Abstract

Little is understood about the long term, indirect health consequences of drought (a period of abnormally dry weather). In particular, the implications of drought for mental health via pathways such as loss of livelihood, diminished social support, and rupture of place bonds have not been extensively studied, leaving a knowledge gap for practitioners and researchers alike. A systematic review of literature was performed to examine the mental health effects of drought. The systematic review results were synthesized to create a causal process diagram that illustrates the pathways linking drought effects to mental health outcomes. Eighty-two articles using a variety of methods in different contexts were gathered from the systematic review. The pathways in the causal process diagram with greatest support in the literature are those focusing on the economic and migratory effects of drought. The diagram highlights the complexity of the relationships between drought and mental health, including the multiple ways that factors can interact and lead to various outcomes. The systematic review and resulting causal process diagram can be used in both practice and theory, including prevention planning, public health programming, vulnerability and risk assessment, and research question guidance. The use of a causal process diagram provides a much needed avenue for integrating the findings of diverse research to further the understanding of the mental health implications of drought.

## 1. Introduction

Drought has been common and widespread in the United States, with perhaps the most notable occurrence being the 1930s drought in the Great Plains, often referred to as the Dust Bowl. This was the US’s most intense drought period to date, with several high-severity droughts occurring in rapid succession such that affected regions could not recover between episodes [1,2]. The 1930s drought lead to widespread agricultural devastation and exacerbated the economic burden of the Great Depression [2]. In the 1950s the Great Plains and southwestern US suffered through another devastating drought, leading to federal drought disaster declarations in 244 of 245 Texas counties [3]. In the late 1980s, the northwestern US and northern Great Plains suffered a significant drought that became the most expensive disaster of any kind to affect the country up to that point, including agricultural damages and damages from wildfires throughout Yellowstone National Park [3,4]. More recently at the peak of the historical drought of 2012, 61.8% of the contiguous US experienced moderate to extreme drought conditions [5]. Continuing into 2015, several West Coast, Southwest, and Southern Great Plains states experienced moderate to severe droughts that led to declared states of emergency, record low lake levels, road-closing dust storms, and wildfires [6,7,8,9,10,11]. Conditions were particularly dire in California, where the governor issued an executive order mandating substantial water reductions in response [12].

### 1.1. Climate Change and Drought

Over the past 50 years, drought frequency and intensity has increased with rising temperatures in much of the Southeast and large parts of the West, and confidence is high that longer-term droughts are expected to intensify in large areas of the Southwest, southern Great Plains, and Southeast [13,14]. There is evidence of shrinking glaciers, decreasing amounts of water in spring snowpack, and shifts to earlier peak flow in snow dominated rivers in western North America [15]. In the most recent assessment report, the Intergovernmental Panel on Climate Change (IPCC) stated with high confidence that water resources are already strained in parts of North America; however, it is not currently possible to attribute the changes is North American drought frequency to anthropogenic climate change [16]. Even so, there is high confidence that water supplies in arid and semiarid western United States are projected to be further stressed by climate change, which will result in amplified water scarcity and drought conditions [13,14,16].

### 1.2. Drought Health Effects

Droughts have been characterized as slow-moving disasters. Like other disasters, droughts often have significant health effects, typically mediated through complex environmental, economic and social pathways. Observed adverse impacts on livelihoods, economic activities, infrastructure, and access to services in North American urban and rural settlements over the past few decades have been at least partially attributed to droughts [16]. Furthermore, given the projections of persistent drought conditions described in both the Fifth Assessment Report of the IPCC and the US National Climate Assessment, there is concern that adverse health outcomes may become more prevalent [13,14,16].

The catalogue of harmful health effects associated with drought is still being assembled and is an area of active study. Several distinct health outcomes have been identified and multiple causal pathways proposed. For example, increased amounts of airborne dust and particulate air pollution can exacerbate asthma, respiratory allergies, and airway disease [17]. Drought conditions can reduce the availability of fresh water, increasing the risk for diseases associated with poor hygiene [18]. Drought can also compromise agricultural production, decreasing food security and threatening livelihoods [19]. From an economic perspective, slow-onset disasters like drought have been found to have a more extensive and destructive impact in the long term than fast-onset disasters [20]. For example, the 2012 drought and heat wave in the US is estimated to have cost approximately $31 billion—one of the country’s most expensive weather disasters [21]. Despite the magnitude of these impacts, the literature on drought health impacts remains relatively thin, particularly in the category of mental health.

### 1.3. Characterization of Exposure

Exposure assessment issues complicate the study of drought health outcomes. Drought onset is difficult to determine: Several dry years in a row may or may not indicate the beginning of a long and significant drought phase [22,23]. Furthermore, environmental changes accumulate over time and can exhibit threshold dynamics, making the consequences of drought not immediately identifiable [24,25,26]. Drought analyses are also complicated by discipline-specific definitions of the hazard and related issues. There are several different ways of measuring drought, including meteorological, hydrological, agricultural and socioeconomic [27]. Meteorological drought is defined based on the duration of the dry period and the degree of dryness in comparison to an average. Hydrological drought is associated with a shortfall of precipitation on surface or subsurface water supply, and is often defined on a watershed or river basin scale. Agricultural drought combines characteristics from meteorological and hydrological drought, such as precipitation shortages, soil water deficits, reduced groundwater, *etc*., and relates them to agricultural impacts. While these first three approaches measure drought in physical terms, socioeconomic drought associates meteorological, hydrological, or agricultural drought with economic measures. Socioeconomic drought occurs when deficient water supply interrupts or decreases the supply of economic goods.

### 1.4. Characterization of Health Outcomes

Defining health outcomes associated with drought is also challenging, particularly in the area of mental health. Interpretations of mental health outcomes vary across studies, and often outcomes are not explicitly defined. Although mental health concepts are complex and vary with social, cultural, and familial norms and values, categorization of adverse mental health outcomes is a prerequisite of further study. It is important to distinguish mental health and mental disorder. According to the World Health Organization, mental health is “a state of well-being in which an individual realizes his or her own abilities, can cope with the normal stresses of life, can work productively and is able to make a contribution to his or her community” [28]. Mental health is more than just the absence of a mental illness or disorder, and it is determined by a host of socioeconomic, biological, and environmental factors. 

According to the Diagnostic and Statistical Manual, 5th edition, of the American Psychiatric Association (DSM-V), mental disorders are characterized by “clinically significant disturbance in an individual’s cognition, emotion regulation, or behavior that reflects a dysfunction in the psychological, biological, or developmental processes underlying mental functioning” [29]. The DSM-V includes extensive diagnostic criteria and codes for mental disorders and is typically used by clinicians to assess patients and aid in developing a comprehensive case formation. Depressive and anxiety disorders are two of the most prevalent mental disorders, and are responsible for approximately 44% of the mental and behavioral health disease burden in the United States [30]. Common symptoms of depressive disorders include the presence of sad, empty, or irritable mood, accompanied by bodily and cognitive changes that significantly affect the individual’s capacity to function [31]. Anxiety disorders are characterized by excessive fear and anxiety, as well as worry about any number of events or activities [32]. Suicide ideation may also be a feature of depressive and anxiety disorders [31,32].

### 1.5. Sub-Acute Disasters and Mental Health

There is a paucity of quantitative epidemiological evidence relating mental health to sub-acute weather disasters like drought [33,34]. Most of the research in the area of climate and mental health has focused primarily on the health effects of acute weather events and natural disasters, such as earthquakes, heat waves, floods, hurricanes, and other storms [35,36,37,38]. While these studies demonstrate increasing attention to the mental health impacts of disasters, there is still much to be discovered about adverse health impacts of non-acute events. Droughts pose a unique threat to mental health with their slow onset, extended exposure windows, and indirect mechanisms of effects, leaving a knowledge gap to be addressed. 

### 1.6. Objectives

Our aim is to examine the direct and indirect pathways linking drought to adverse mental health outcomes. Our objectives are to:

1. Systematically review the existing literature concerning drought and mental health outcomes.
2. Use systematic review results to develop a causal process diagram depicting the pathways linking drought and mental health outcomes.
3. Examine risk and protective factors associated with drought exposure and mental health outcomes, including the coping mechanisms used by populations experiencing drought.

Synthesizing the research and developing a framework to illustrate the role that drought has on mental health will enable a better understanding of the complexities between specific components of this relationship, as well as highlight areas in need of further research. Additionally, examining the vulnerabilities that make individuals more susceptible to negative mental health outcomes will bring attention to those who need it the most while also shedding light on possible avenues for intervention.

## 2. Methods 

To address objective 1, a systematic review process was adapted from those described in Khan*, et al.* [39], Hosking and Campbell-Lendrum [40], and Hess*, et al.* [41]. To capture as many relevant citations as possible, keyword/phrase searches were conducted during January 2015 in PubMed, PsychINFO, Web of Science, and Google Scholar. Each database was searched for following terms: “drought” and (“mental health” or “suicide” or “depression” or “anxiety” or “emotional distress” or “psychological distress” or “schizophrenia” or “bipolar” or “manic depression”). Titles and abstracts of the resulting citations were first reviewed to determine relevance to the project. Only peer-reviewed journal articles about human response to aspects of extreme weather events were included in this study. Review articles were excluded unless they added novel information to the body of knowledge, such as a meta-synthesis or the new application of a theoretical framework. The full papers of the remaining citations were assessed to select for continued inclusion those studies that related to a major component of a causal pathway linking drought to a human mental health response. All determinations were made by HV in consultation with the other authors.

To address objective 2, a causal process diagram using information from the systematic review was created to depict the numerous complex links in the drought/mental health relationship. Causal process diagrams are visual representations of the way in which interacting factors behave within a complex system. They are not only useful for summarizing and organizing information from interdisciplinary research, but are also logically rigorous and can help with planning data collection and analysis [42]. Our goal was to develop a causal process diagram to synthesize existing knowledge surrounding the mental health outcomes of drought and provide guidance about future research directions. The diagram was constructed following the recommended approach for developing and reporting a causal process diagram as described in Joffe and Mindell [42] and expands upon previous frameworks proposed by Perry [43] and Berry, Bowen and Kjellstrom [33]. Mental health outcomes were grouped in the diagram according to similar symptoms listed in the Diagnostic and Statistical Manual of Mental Disorders (DSM-V).

To address objective 3, a table was created using information from the systematic review that focused on specific factors that may influence how susceptible a person or larger group is to the exposures detailed in the causal process diagram. We also included several coping mechanisms found in the literature.

## 3. Results 

### 3.1. Study Characteristics 

**Figure 1:**
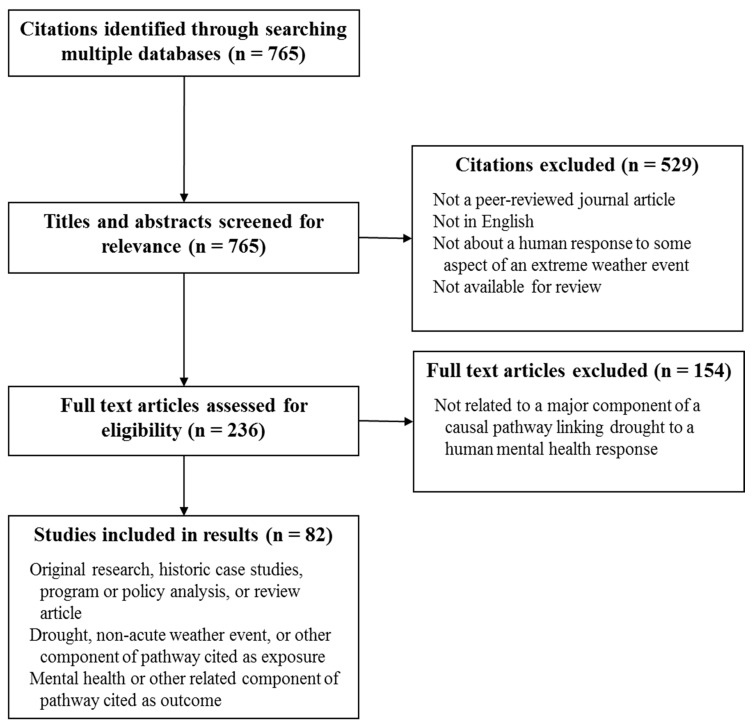
Flow chart of article inclusion and exclusion.

Of the 765 studies initially identified, 82 were eligible for inclusion in the final review. Figure 1 contains a flow chart of article inclusion and exclusion. The majority of studies involved original research and were from developed nations, with Australia being the most common study locale. Eighty studies contributed information in support of links in the diagram, while two studies found no association. A full summary of the study characteristics can be found in Table 1. 

It is important to note that not all of the papers included in the final review specifically analyzed mental health outcomes of drought. However, articles were included if there were discussions of mental health outcomes in the context of populations who are vulnerable to drought. Oftentimes these papers looked at specific coping behaviors or protective factors related to mental health. These types of results were included in a separate table (Table 2) meant to supplement the causal process diagram. 

### 3.2. Causal Process Diagrams

**Figure 2:**
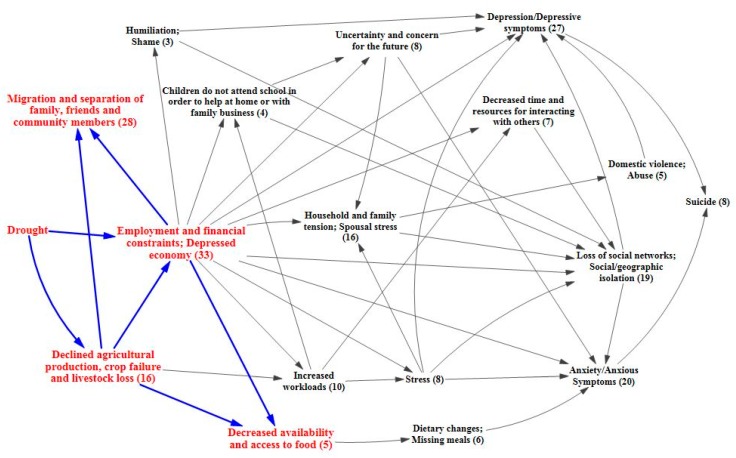
Direct economic effects of drought.

The following causal process diagrams highlight major pathways identified in the literature through which drought has been linked with adverse mental health outcomes. Some pathways have greater evidentiary support than others, as indicated by a greater number of supporting articles found through the systematic review. In each of the causal process diagrams, the number within the parenthesis indicates how many articles were found to support that step in the pathway. Of greatest support is the pathway focusing on the economic effects of drought, with a total of 33 papers of support. Drought can adversely impact individual and community economic activities, particularly those with livelihoods influenced by weather conditions and water access [44,45,46,47,48,49,50,51,52,53,54,55,56,57]. The agricultural sector is typically hit hardest, and farmers can experience declined production, crop loss, and livestock failure [34,58,59,60,61,62,63,64]. These losses can lead to financial constraints and unemployment, as well as issues associated with food availability and access [24,61,63,64,65,66,67,68,69]. Migration away from the drought prone area, often in search of better employment opportunities, is a related outcome [56,60,64,70,71,72,73,74,75,76]. Figure 2 highlights the direct economic effects of drought. 

**Table 1:** Summary of Characteristics of Included Studies.

	Characteristic	No. of Eligible Studies	Percent of Total	Percent of Subcategory
Study Type	Original research	53	65%	-
	*Quantitative methods*	24	-	45%
	*Qualitative methods*	21	-	40%
	*Mixed methods*	8	-	15%
	Historic case studies	8	10%	-
	Program/Policy analyses	5	6%	-
	Review articles	16	20%	-
Study Location	United States	7	9%	-
	Other developed countries	58	71%	-
	*Australia*	52	-	90%
	*Canada*	6	-	10%
	*New Zealand*	1	-	2%
	*United Kingdom*	1	-	2%
	Developing countries	10	12%	-
	*Bolivia*	2	-	20%
	*Botswana*	1	-	10 %
	*Brazil*	1	-	10%
	*Ethiopia*	1	-	10%
	*India*	4	-	40%
	*Iran*	1	-	10%
	Not location specific	7	9%	-
Publication Date	Articles published 2005 or later	72	90%	-
	Articles published 1996–2004	6	7%	-
	Articles published 1995 or earlier	4	5%	-

Percent totals may not sum to 100% due to rounding.

**Figure 3:**
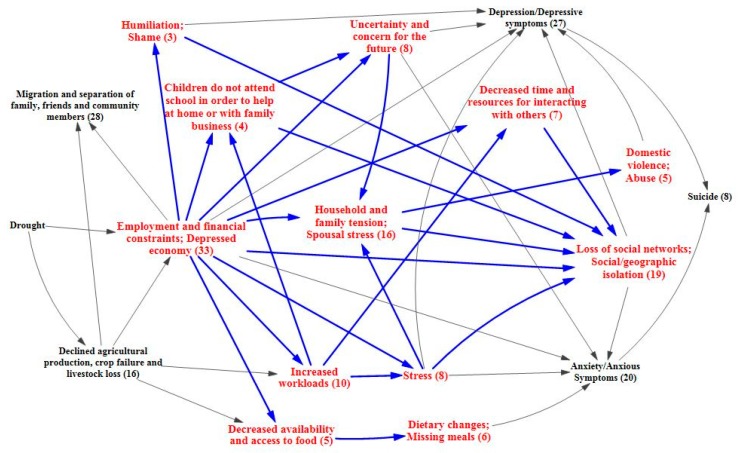
Intermediary factors of economic pathway.

**Figure 4:**
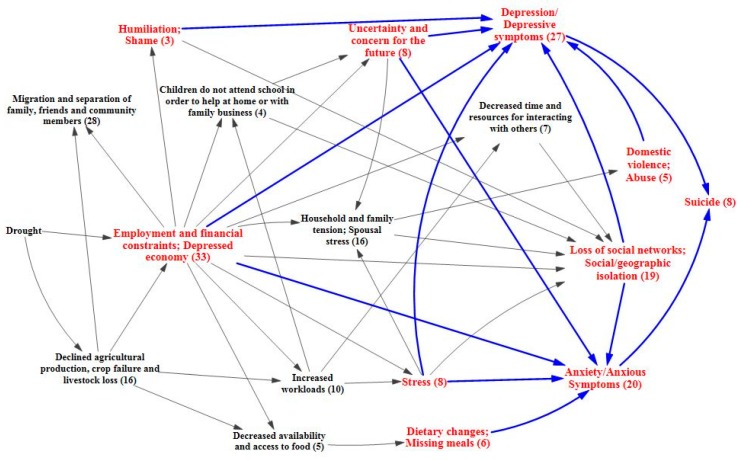
Mental health outcomes of economic effects of drought.

While individuals will react to financial hardship in different ways, there is evidence that stress and social isolation may occur, as well as the possibility of increased workloads, decreased time and resources, and disruption of children’s education so that they can help at home or with the family business [44,45,48,50,53,58,67,77,78,79,80]. These stressful situations cause uncertainty about the future, increasing anxiety [44,50,51,56,58,67,81]. Shame and humiliation over financial struggles may also contribute to social isolation and depressive symptoms [46,67,82]. Additionally, there is evidence that these types of situations allow stress and tension to permeate the household, sometimes resulting in domestic abuse [44,46,48,50,58,66,67,70,81,83,84]. These economic-related pathways overlap and interact in such a way that they may result in depression, anxiety, and suicide [53,61,62,76,78,79,81,85,86]. Evidence also shows that the more severe the drought and its impacts upon livelihoods, the larger the negative impacts upon the mental health for those affected [54,62,87]. The intermediary factors and outcomes associated with the economic pathway are shown in Figure 3 and Figure 4, respectively.

**Figure 5:**
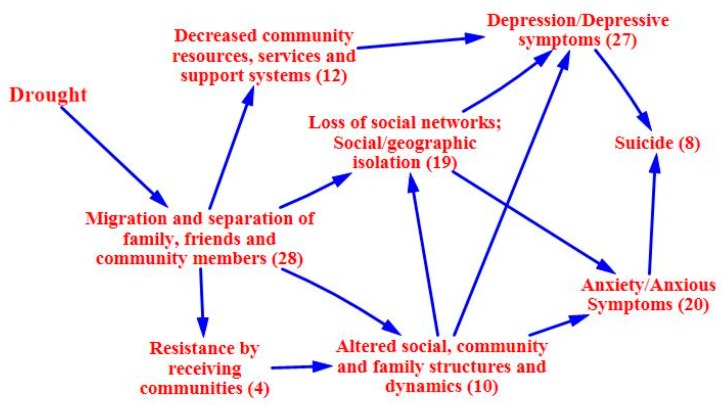
Migratory effects of drought upon mental health.

Looking at the pathway from a different angle, the migratory effects of drought also have implications for mental health that are well supported in the literature, with the systematic review identifying 28 papers related to this pathway. Migration away from drought-stricken communities with depressed economies can lead to a reduction in community resources, services, and support systems [45,52,54,56,61,70,76,88]. Altered family and community structures coupled with social and geographical isolation can also be issues for those who emigrate and those left behind, and may lead to symptoms of anxiety and depression [50,53,58,67,72]. Poor reception of immigrants by receiving communities has been observed [73,89,90,91]. A feedback loop may be of particular concern in this situation, with drought spurring migration and contributing to the erosion of the resource base, which in turn amplifies the effects of drought upon the community and pushes more people to leave. In such a case, a lack of resources is not only caused by drought, but is also an issue that makes drought more difficult to cope with [56]. Figure 5 focuses on the migratory effects pathway.

**Figure 6:**
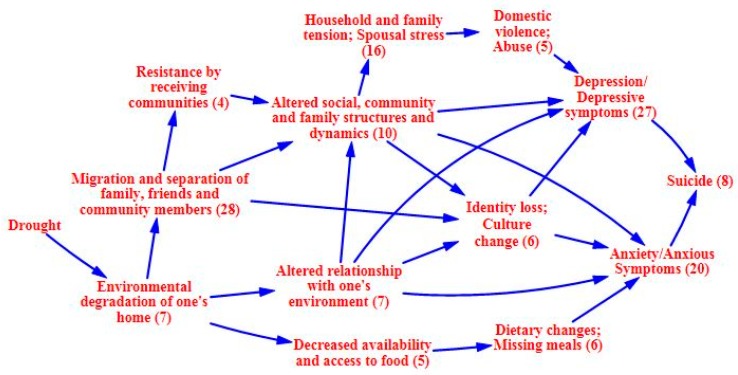
Mental health outcomes related to the environmental degradation of one’s home.

There is also evidence that drought can lead to environmental degradation of one’s home, affecting people’s ability to interact with their environment and causing additional issues related to rupture of place bonds, culture change and loss, and altered community and family dynamics [49,70,89,91,92,93,94]. These are particular issues for those who gain a strong sense of identity from the land and take on an environmental stewardship role [55,69,84,88]. Environmental degradation can also lead to changes in local and regional plants and wildlife, which may have downstream impacts for food security [63,69]. Such alternations in one’s way of life can contribute to depression and anxiety for individuals and tension within family and social networks [56,69,84,88,92]. The effects of environmental degradation of one’s home are depicted in Figure 6.

**Figure 7:**
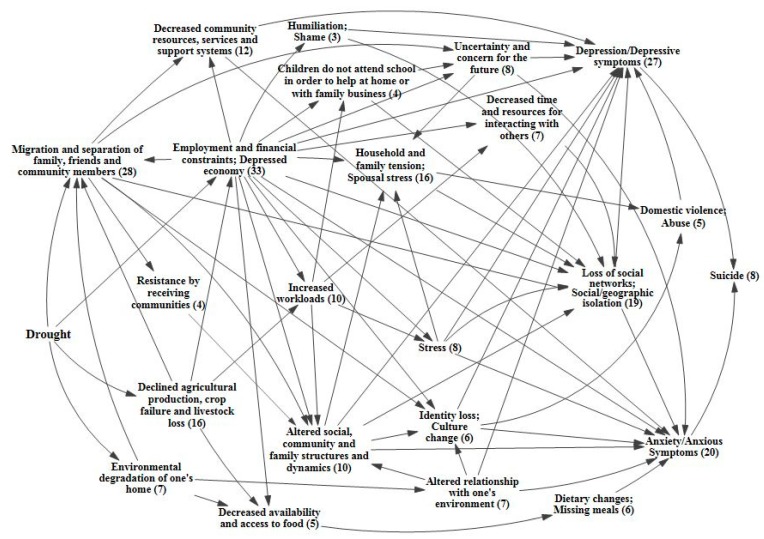
Causal processes diagram for mental health outcomes of drought.

The preceding diagrams on the economic, migratory and environmental degradation effects of drought can be combined into one overarching causal process diagram (Figure 7) that shows how all of these individual components relate and overlap with one another. This highlights the highly interactive nature of the direct and indirect effects of drought and their resulting impact upon mental health.

### 3.3. Vulnerabilities, Protective Factors, and Coping Strategies

Certain populations experience heightened risk of adverse mental health impacts from drought, while others have distinct protective factors and potential coping strategies. Important vulnerable populations, coping mechanisms, and protective factors found in the literature are presented in Table 2. 

As the diagram illustrates, there are a variety of different mechanisms through which drought can affect individuals, with certain populations being more vulnerable than others. For example, rural and remote populations face a unique set of challenges that increase their vulnerability. They are thus frequently studied; the systematic review found 32 articles that focused on rural individuals and communities. The circumstances of rural communities sometimes cause health to be defined differently, often with an emphasis on one’s ability to be productive and with distress seen more as a problem of daily living rather than a mental health issue [77,95]. Research has repeatedly demonstrated a phenomenon of rural stoicism that, combined with a culture of self-reliance, can interfere with help-seeking behaviors and limit effective adaptation to changed circumstances [44,77,96,97]. The social visibility present in small, rural communities can exacerbate reluctance to seek assistance for mental health problems [79,96,97,98,99]. Individuals who may consider pursuing mental health services are afraid of marginalization if others find out. Finally, compounding all of these problems is the lack, or perceived lack, of community resources and infrastructure in remote areas [44,47,56,71,75,95,98,100]. If individuals do not believe there are mental health services available to them, these resources will not be utilized.

**Table 2:** Vulnerabilities, protective factors and coping mechanisms for mental health effects of drought.

Vulnerable Characteristics	Protective Factors	Coping Mechanisms
Rural or remote population (32) Farming or agriculture dependent population (22) Indigenous population (6) Perceived stigma associated with mental health issues (12) Lack of knowledge surrounding mental health issues and availability of services (11) Previous mental health issues or other adverse life events (5) Exposure to an extended period, more intense or severe drought (5)	Social support, social capital and sense of community belonging (15) Shared knowledge and community preparedness regarding availability and access to services (6) Mental health literacy (2) Government assistance and initiatives in place (6)	Employing practical solutions and active methods, including planning for the future (7) Psychological methods, including positive thinking, acceptance and reframing of the problem (4) Utilizing social support and talking about the problem (3) Distracting oneself from the problem; taking a break (2) Turning to religion (3) Alcohol and substance use (8) Denial and avoidance of the problem (3)

Number within parenthesis indicates how many articles were found in the review to support that characteristic, mechanism or factor.

Rural communities often include farming populations, another well-studied group. The systematic review revealed 22 articles that focused on the mental health outcomes of farmers and their families. Farming populations face many of the same challenges described for rural and remote populations, including declining population density, a lack of mental health services, and stigma surrounding mental health issues. Added to these issues is the fact that drought directly impacts the employment and economic success of agricultural communities [26,47]. Rural male farmers, who are particularly well studied, have several distinct risk factors [44,45,101,102,103]. Masculine hegemony dictates that men be emotionally tough, stoic in the face of adversity, and the breadwinners of the family [44,45,53]. These normative stereotypes harm help-seeking behaviors. Furthermore, the stigma associated with mental health issues is reinforced by the paradigm of masculinity found in farming [47,75,99,101,103]. Similarly, some studies of women in rural and farming communities have found heightened vulnerability due to their roles as caregivers and household managers, and the additional stresses associated with those responsibilities [48,95,96]. However, complicating this finding are the results of two studies that found either no heath effect or declined risk of suicide for females as drought increases [87,104]. This indicates there may be a more complex causal pathway involving gender, which modifies the effects of drought. 

Due to their often strong connection with the land, indigenous groups are also particularly vulnerable [47,55,69,84,95]. Sustaining a relationship with the land in the face of drought is challenging, and as the climate continues to change, individuals will likely have a harder time maintaining their way of life [84]. This can lead to acculturation by forcing people to change their traditional ways of living and make individuals more vulnerable to mental health issues [93].

While the geographic and social isolation of rural and farming communities can increase vulnerability, having a strong sense of belonging, maintaining social capital, and utilizing those connections can serve as protective factors and coping mechanisms [43,56,100,102,103,105,106,107]. Protective social interactions can be at an individual or communal level [25,101]. Oftentimes small, rural populations are able to maintain a sense of community and informal support networks that can be utilized to address mental health issues; however, as drought conditions worsen these ties are strained and can break down due to prolonged pressure on community resources [70,101,108,109]. 

Practical solutions like farming adaptations were often cited as a way to cope with drought difficulties. Historically, farmers have changed farming practices during times of drought, and innovations such as land diversification and farm expansion are still used today [60,71,108]. Economic and lifestyle planning such that retirement and future generations would not be jeopardized by a failing farm was another way individuals were able to practically cope with drought [108,110]. 

However, not all coping mechanisms confer long-term advantage. In some situations avoidance and denial of the problem is common [56,91,108]. Although it is not always the main strategy, substance and alcohol abuse were cited in several studies as a way in which individuals dealt with the stressful situations caused by drought [44,58,84,85,107], particularly among males [81,107,111]. 

Mental health literacy has been identified as a protective factor and a way to reduce stigma by increasing knowledge and increasing help-seeking behaviors [97]. Providing information about mental health problems and services is also an important way to empower individuals to get through tough times [25,101]. Finally, the presence of government initiatives to assist during times of drought, either financially or through mental health outreach programs, may be protective as well [25,55,71,97,112]. However, it is important for such initiatives to be easily navigable and culturally appropriate [79,97].

## 4. Discussion

There is a growing need for public health practitioners and researchers to understand drought’s health impacts. Our review suggests several pathways for drought to adversely affect mental health, particularly among vulnerable populations. Some pathways were supported by more evidence than others. The economic effects pathway, particularly its impacts on rural farming populations, as well as the migration pathway, were well supported. The environmental degradation pathway had less support. This pathway is perhaps most relevant to indigenous populations, a potentially vulnerable group, and more research should be done to examine the strength of the postulated relationships. 

The retrieved literature used a variety of methodologies. Qualitative and quantitative methods were used with nearly the same frequency, while a smaller portion used a mixed methods approach. Choice of methodology is important when looking to develop a detailed understanding of a complex phenomenon. Typically, qualitative methods are able to collect rich data that provide an in-depth understanding of research issues that embrace the perspectives of the study population and the context of their specific demographics. Quantitative methods have the advantage of allowing for numeric risk estimates that can be used to perform scenario-based health impact projections, which have become a mainstay of climate-health research. There was little quantitative estimation of the risks drought poses for the incidence of adverse mental health effects in the literature identified. In trying to develop an understanding of the casual relationship between drought and mental health that can be meaningful for a wide range of individuals, mixed methods approaches offer the ability to take advantage of the strengths of both quantitative and qualitative approaches. 

There was a high degree of concordance among the identified literature leaving a strong impression of increased risk for adverse mental health impacts associated with drought. However, there were studies found in the systematic review that did not lend support to some of the relationships postulated in other literature. Guiney [113] examined intentional self-harm deaths of farmers in relation to prolonged droughts in Victoria, Australia and found no pattern of increasing farming suicides during the drought years. The findings of study are hampered though because it did not include a non-drought comparison to serve as a baseline for comparison. Furthermore, the paper does not account for the Australian gun control policy which was introduced at the start of the study period and is credited with reducing suicide risk by a large amount [114]. The author also acknowledges that community support programs targeting drought-affected areas may have contributed to improved outcomes during the time period in question. In another study, Powers *et al.* [104], found that adverse climate events did not appear to affect Australian women’s health and well-being. This relationship has been confirmed across other studies and complicates the proposed relationship between drought and mental health [87,115]. However, occupation is not explicitly examined in these studies, which may play an important role in the female experience of drought. Further study of the posited relationship with a focus on gender and occupation is therefore warranted. It is also worth noting that Powers *et al.* [104] suggest that the multiple resources available in high income counties, as well as tendencies for preparation and adaptation, may be responsible for mitigating some of the health impacts of adverse climate events like drought. This upholds the assertion that community support and preparedness may be able to offset the negative mental health impacts of drought. 

This study had several limitations. First, the systematic review may have inadvertently missed publications, particularly those in the grey literature. Second, the relationships diagrammed are limited to those identified in the literature, though there are other ways in which drought might affect mental health. Third, physical health effects and their relation to mental health are underrepresented in the diagram, leaving questions about their role in the causal process. Fourth, some of the findings may not be generalizable, particularly due to cultural differences in the definitions of mental health. Lastly, it is also possible that unexamined factors may complicate the associations posited in the diagram. One plausible example of this could be pesticide exposure, which could act as a causal intermediary or confounder depending on whether pesticides are part of the proposed causal pathway under study. Drought may increase the use of pesticides or change people’s exposure to them, and these could have physical and neuroendocrine effects that could have implications for mental health [116,117]. However, pesticide exposure was not explicitly considered in the studies collected by the systematic review. A future study looking at the mental health effects of drought among a farming population using pesticides should consider whether pesticides may be part of the causal pathway under study and, if not, treat pesticide exposure as a potential confounder. 

### 4.1. Application of the Causal Process Diagram

A note of caution related to linear interpretation of the causal diagram is in order. Perry [43] wrote that simple, uni-causal models are not particularly useful for understanding the physiological consequences of natural disasters. Instead, disasters should be seen as a contributory cause—one of a number of factors that together determine psychological consequences. Keeping this in mind when considering the relationships between drought and mental health underlines the importance of multiple pathways and nodes as much as the linear process suggested by the different pathways. 

Despite its limitations, the causal process diagram has several potential applications, including prevention planning, public health programming, vulnerability and risk assessment, and research question development.

#### 4.1.1. Prevention Planning

The diagram can be used by public health practitioners or extension workers in order to look for upstream warning signs of mental health issues associated with drought. This could be particularly relevant in the case of feedback loops, where there is interplay between drought-related stressors, coping or adaptation strategies, and downstream impacts. For example, in the current California drought, farmers have taken to pumping groundwater to cope with surface water losses [118]. As groundwater levels deplete, pumping costs will increase and may be subject to limits [118]. This could further threaten agricultural production and amplify drought-related stressors. The causal diagram can help identify strategies that anticipate such feedbacks and thereby promote adaptive strategies over short-term coping efforts.

**Table 3:** Suggested Data Sources for Future Research.

Broad Category	Component of Diagram/Table	Data	Available from
Distal Exposure	Drought	Palmer drought severity and crop moisture indices for the US	National Weather Service/NOAA Center for Weather and Climate Prediction
	Drought	GIS and tabular data for drought severity the United States	United States Drought Monitor
Secondary and Intermediate Exposures	Declined crop production, crop failure and livestock loss	Various datasets pertaining to crop production, food prices, and farm economies	United States Department of Agriculture
	Declined crop production, crop failure and livestock loss	Agricultural data searchable by commodity, location or time period	United States Department of Agriculture
	Migration	Changing population counts in rural and urban areas	United States Census
	Employment and financial constraints/depressed economy	Unemployment and labor force statistics	Bureau of Labor Statistics
	Employment and financial constraints/depressed economy	Poverty data	United States Census
	Children do not attend school	School enrollment data	United States Census
Protective Factors and Coping Strategies	Social support	Data on available mental health treatment and levels of emotional support available	Health Indicators Warehouse—Department of Health and Human Services
	Alcohol and Substance Use	Data on alcohol and illicit drug usage	Health Indicators Warehouse - Department of Health and Human Services
Outcomes	Mental Health	Data on anxiety, depression, conduct and panic disorders	National Health and Nutrition Examination Survey—Centers for Disease Control and Prevention
	Mental Health	Data on mental health disorders, suicide attempts and deaths	Health Indicators Warehouse—Department of Health and Human Services
	Mental Health	Data on depressive disease, anxiety, suicide, domestic violence	Emergency department records
	Mental Health	Suicide as cause of death data	National Violent Death Reporting System—Centers for Disease Control and Prevention

#### 4.1.2. Public Health Programming

The diagram offers a useful approach to examining interactions among stressors and impacts, which could be valuable when developing programs or interventions for communities. The literature review revealed that several mental health initiatives related to drought have already been implemented across the globe with varying degrees of success [25,34,43,52,68,80,85,112]. Adapting successful interventions using the causal process diagram could be another practical use of this research. 

#### 4.1.3. Vulnerability and Risk Assessment 

Public health has often used environmental risk assessment to estimate human exposure to harmful or toxic substances; these methods have been expanded over recent years to estimate adverse effects of climate change, as seen in the methods used by the Intergovernmental Panel on Climate Change [119]. This concept of analyzing possible impact from an environmental change can be applied to causal process between drought and health. The diagram and complementary table highlight areas of exposure and characteristics that make individuals particularly vulnerable to drought. This information can be used to inform vulnerability and risk assessments, allowing for the characterization of risk to the exposed populations. 

#### 4.1.4. Future Research

In addition to practical applications, the diagram can be used to generate research questions exploring the mental health impacts of drought effect and potential interventions for averting or mitigating these impacts. By providing a way to conceptualize causal processes, the diagram can both inform and guide research. To assist in this approach, a list of available data sources have been compiled that can be used to quantitatively explore the posited relationships in the causal process diagram (Table 3). Much of this data is publically available, and the list is not exhaustive. The focus is primarily on data relevant to US populations, as this is the context with which the authors are most familiar, and there are likely other data sources in other regions that are worth exploring. 

## 5. Conclusions 

Given the current drought situation affecting parts of the United States and increasing concern over the association between climate change and drought, the linkages between drought and mental health are increasingly important. There is a substantial body of literature on the topic that allows for identification of several distinct and inter-related pathways by which drought can adversely impact mental health as well as several coping and adaptation strategies. Most of these relationships are mediated through environmental or economic pathways, and the outcomes most closely studied are mood disorders and, to a lesser degree, intimate partner violence and suicide. Few associations between drought exposure and adverse mental health outcomes have been quantified. This research is an initial step in bringing this important issue forward and outlining possible implications for prevention, mental health services, and future research.
